# Engineering a robust cell-surface display platform in the multi-stress-tolerant yeast *Issatchenkia orientalis*

**DOI:** 10.1007/s00253-026-13867-1

**Published:** 2026-05-19

**Authors:** Yoshiaki Kawahara, Ryo Nasuno, Yong-Su Jin, Tomohisa Hasunuma

**Affiliations:** 1 Graduate School of Science, Technology and Innovation, Kobe University, 1-1 Rokkodai, Nada, Kobe, 657-8501 Japan; 2 Engineering Biology Research Center, Kobe University, 1-1 Rokkodai, Nada, Kobe, 657-8501 Japan; 3 Department of Food Science and Human Nutrition, University of Illinois at Urbana-Champaign, Urbana, IL 61801 USA; 4 Carl R. Woese Institute for Genomic Biology, University of Illinois at Urbana‐Champaign, Urbana, IL 61801 USA

**Keywords:** Yeast cell-surface display, *Issatchenkia orientalis*, Lignocellulosic biomass, Multi-stress tolerance, β-glucosidase

## Abstract

**Abstract:**

Displaying cellulolytic enzymes on the yeast cell surface via the anchoring domain of glycosylphosphatidylinositol-anchored proteins can integrate enzyme production, saccharification, and fermentation into a single step in biomass fermentation. Therefore, this technique represents a promising strategy for cost-effective and sustainable production of value-added chemicals and biofuels from lignocellulosic biomass (LCB), the most abundant renewable resource, which typically requires multiple complex processing steps. However, pretreatment of LCB, such as acidic thermochemical treatment, causes harsh cultivation conditions characterized by low pH, high temperature, and lignocellulosic fermentation inhibitors. Thus, host strains displaying cellulolytic enzymes must exhibit remarkable tolerance to these stresses. Here, we employed the non-conventional yeast *Issatchenkia orientalis*, which has remarkable multi-stress tolerance, and successfully established the display system in this yeast. We found that the cell-surface display cassette that functions in the model yeast *Saccharomyces cerevisiae* was not functional in *I. orientalis*; therefore, we constructed a modified display cassette based on the *I. orientalis SED1* gene. Using this cassette, we successfully displayed fluorescent protein and β-glucosidase (BGL) on the cell surface. The BGL-displaying strain grew robustly on cellobiose as the sole carbon source and, notably, maintained growth even in the presence of lignocellulosic fermentation inhibitors. To the best of our knowledge, this is the first report demonstrating the successful immobilization of functional proteins on the cell surface of *I. orientalis* and assimilation of LCB-derived intermediates, even under stress conditions, by displaying cellulolytic enzymes, highlighting its potential as a general industrial platform for sustainable LCB bioconversion.

**Key points:**

- *A cell-surface display cassette for Issatchenkia orientalis was constructed.*
- *The β-glucosidase-display strain utilized cellobiose as the sole carbon source.*
- *Cellobiose assimilation was observed even in the presence of fermentation inhibitors.*

**Supplementary Information:**

The online version contains supplementary material available at 10.1007/s00253-026-13867-1.

## Introduction

Global challenges such as increasingly severe climate change and the depletion of petroleum resources have accelerated demand for sustainable production systems that are independent of fossil-based feedstocks. Lignocellulosic biomass (LCB), including rice straw, corn stover, wood chips, bagasse, and other agricultural residues, is one of the most promising alternatives owing to its abundant availability and non-competition with food and feed crops (Sharma et al. 2022). LCB is primarily composed of three structural polymers: cellulose, hemicellulose, and lignin. Among these, cellulose and hemicellulose are polysaccharides that typically account for 40–50% and 20–40% of the total biomass, respectively (McKendry 2002). These polysaccharides can be hydrolyzed into fermentable monosaccharides such as glucose, xylose, arabinose, and galactose through chemical and enzymatic treatments, and the released sugars are subsequently converted to bio-based chemicals and fuels by metabolically engineered microorganisms.

In LCB bioconversion, yeasts are promising host organisms due to several advantages, including resistance to bacteriophage infection, ease of genetic manipulation, and a high capacity for heterologous protein expression (Espinosa et al. 2020; Gomes et al. 2018; Idiris et al. 2010). However, most model yeasts, such as the budding yeast *Saccharomyces cerevisiae*, lack the native ability to hydrolyze cellulose and hemicellulose into monosaccharides, necessitating the introduction of heterologous hydrolytic enzyme genes. To address this issue, the cell-surface display system has emerged as one of the most promising approaches and has been extensively studied for decades (Minnaar et al. 2024; Zhang et al. 2022). In this technique, functional proteins, such as hydrolytic enzymes, are fused to anchoring proteins, typically glycosylphosphatidylinositol (GPI)-anchored proteins, and immobilized on the cell surface. The surface-displayed hydrolytic enzymes degrade extracellular polysaccharides, and the released monosaccharides are simultaneously taken up and fermented by the host. Therefore, this system integrates several steps of LCB bioconversion, such as saccharification, enzyme production, and fermentation, into a single streamlined process, thereby simplifying the overall bioconversion and reducing production costs (den Haan et al. 2015; Hasunuma and Kondo 2012).

For efficient LCB utilization, pretreatment, such as acidic thermochemical treatment, is required to disrupt its rigid crystalline structure (Jönsson et al. 2013). However, such treatment simultaneously generates harsh cultivation conditions, including extremely low pH, high temperature, and the presence of lignocellulosic fermentation inhibitors such as acetic acid, formic acid, furfural, 5-hydroxymethylfurfural (5-HMF), and phenolic compounds, all of which hinder microbial growth and fermentation (Jönsson and Martín 2016; Palmqvist and Hahn-Hägerdal 2000). Therefore, host yeast strains must exhibit high tolerance to these stresses. To date, various strategies have been employed to overcome these stressful conditions, including clarification of stress response mechanisms (Palmqvist and Hahn-Hägerdal 2000; Mira et al. 2010; Verghese et al. 2012), genetic manipulation of endogenous genes (Yang et al. 2023; Swinnen et al. 2017), introduction of heterologous genes (Matsushika et al. 2016; Wang et al. 2017, 2024), and the use of inherently stress-tolerant yeasts as alternative host organisms, such as the thermotolerant yeast *Kluyveromyces marxianus* (Yanase et al. 2010). Although many previous engineering efforts have focused on tolerance to a single stress (Hemansi et al. 2022; Li et al. 2022), the development of host strains exhibiting the robust multi-stress tolerance required for LCB bioconversion, particularly tolerance to low pH, high temperature, and lignocellulosic fermentation inhibitors, has remained limited.

Recently, the non-conventional yeast *Issatchenkia orientalis*, also known as *Pichia kudriavzevii*, has emerged as a promising host for LCB bioconversion owing to its remarkable multi-stress tolerance (Kwon et al. 2011; Frousnoon et al. 2025; Tan et al. 2025). This yeast grows under strongly acidic conditions, tolerating pH values as low as 1.5 (Dubinkina et al. 2024). In addition, it can grow in the presence of lignocellulosic fermentation inhibitors (Kwon et al. 2011; Zwirzitz et al. 2021) and under high-temperature conditions (Kitagawa et al. 2010; Li et al. 2021). Furthermore, molecular genetic tools for this yeast have been developed rapidly in recent years, including CRISPR/Cas9-mediated genome editing and libraries of constitutive promoters with varying expression strengths (Cao et al. 2020; Fatma et al. 2023). Using these tools, several studies have reported engineered strains capable of high-level production of organic acids, such as succinic and lactic acids (Tran et al. 2023; Lee et al. 2024). Despite its advantageous multi-stress tolerance, *I. orientalis* lacks the intrinsic ability to hydrolyze cellulose and hemicellulose into monosaccharides and requires the introduction of heterologous hydrolytic enzyme genes for effective LCB bioconversion (Kitagawa et al. 2010). The application of cell-surface display systems to *I. orientalis* has not yet been reported.

In this study, we attempted to establish the cell-surface display system for the multi-stress-tolerant yeast *I. orientalis*. We found that the previously established cell-surface display cassette used for the model yeast *S. cerevisiae* did not function in this yeast. Therefore, we constructed a modified display cassette using genetic elements derived from the *I. orientalis SED1* gene, which successfully displayed target proteins on the cell surface. Furthermore, the *Aspergillus aculeatus* β-glucosidase 1 (BGL1)-displaying strain exhibited robust growth on cellobiose as the sole carbon source and, notably, maintained its ability to assimilate cellobiose even in the presence of the fermentation inhibitors, highlighting its potential as a promising industrial platform for microbial bioconversion using LCB.

## Materials and methods

### Strains and media

*Escherichia coli* NovaBlue strain (Novagen, WI, USA) was used as the host for recombinant DNA manipulation. *E. coli* transformants were grown in Luria-Bertani medium (10 g/L of tryptone, 5 g/L of yeast extract, and 5 g/L of sodium chloride) supplemented with 100 µg/mL of ampicillin at 37 °C. The wild-type strains of *I. orientalis* used in this study were 11 strains obtained from NITE Biological Resource Center (NBRC, Tokyo, Japan), and the wild-type strain of *S. cerevisiae* used in this study was BY4741 (Life Technologies, CA, USA). The genetic properties of all used yeast strains and the transformants are summarized in Supplementary Table S1. The medium used for the growth of yeasts was yeast extract peptone dextrose (YPD) (10 g/L of yeast extract, 20 g/L of Bacto-peptone [Difco Laboratories, MI, USA], and 20 g/L of glucose), synthetic complete (SC) dropout medium lacking uracil (SC-URA) (6.7 g/L of yeast nitrogen base without amino acids [Difco Laboratories], 1.92 g/L of yeast synthetic dropout medium supplements without uracil [Sigma-Aldrich, MO, USA], and 20 g/L glucose), or synthetic dextrose (SD) medium (6.7 g/L of yeast nitrogen base without amino acids [Difco Laboratories] and 20 g/L glucose).

### Stress tolerance analysis of *I. orientalis* NBRC1664

To analyze tolerance to low pH and high temperature, *I. orientalis* and *S. cerevisiae* cells were pre-cultured in 3 mL of YPD medium at 30 °C and 200 rpm for 48 h and then inoculated into 5 mL of SC medium with an initial optical density at 600 nm (OD_600_) of 0.05. The initial pH of SC medium was adjusted to 2 or 6 using 1 M HCl and 0.5 M KOH, respectively. Cultivation was performed in L-shaped tubes at 30 °C or 45 °C and 40 rpm for 72 h using a Bio-photorecorder TVS062CA (ADVANTEC, Tokyo, Japan). To evaluate tolerance to lignocellulosic fermentation inhibitors (furfural, 5-HMF and vanillin), the cells were pre-cultured in 3 mL of YPD medium at 30 °C and 200 rpm for 24 h. Serial dilutions of the culture (OD_600_ of 1, 0.1, 0.01, or 0.001) were prepared using Mill-Q water. Then, 3 µL of each dilution was spotted onto SD agar plates supplemented with uracil, leucine, histidine, methionine, and one of the inhibitors (furfural 21 mM; 5-HMF 28 mM; vanillin 10 mM). The plates were incubated at 30 °C for 96–120 h, and colony growth was recorded using a gel documentation system FAS-X (Nippon Genetics, Tokyo, Japan).

### Generation of the uracil-auxotrophic strain of *I. orientalis* NBRC1664

Genomic DNA of *I. orientalis* NBRC1664 was extracted using the Kaneka Easy DNA Extraction Kit version 2 (Kaneka, Osaka, Japan). The upstream (543 bp) and downstream (279 bp) homology arms of the *I. orientalis URA3* (*IoURA3*) open reading frame (ORF) were amplified by polymerase chain reaction (PCR) using the URA3-UP-F and URA3-UP-R primers and URA3-DOWN-F and URA3-DOWN-R primers, respectively (Xiao et al. 2014). These fragments were fused by overlap extension PCR, and the resulting DNA fragments were introduced into NBRC1664. Transformants were selected on SC agar plates supplemented with 1 mg/mL 5-fluoroorotic acid (5-FOA). Deletion of the *IoURA3* ORF was confirmed by colony PCR using the Colony-P-F and Colony-P-R primers. To verify uracil auxotrophy, the transformants were streaked on SD agar plates with or without uracil (76 µg/mL) and incubated at 30 °C.

### Construction of cell-surface display cassettes

All plasmids were constructed using In-Fusion kit (TaKaRa Bio, Shiga, Japan) following the manufacturer’s instructions. The plasmids and primers used in this study are summarized in Supplementary Tables S2 and S3, respectively.

The DNA fragment of the *IoURA3* gene was amplified from *I. orientalis* NBRC1664 genomic DNA by PCR using the URA3-F and Colony-P-R primers. The amplified fragment was introduced into the *Hind*III site of the yeast centromeric shuttle vector pRS415, resulting in the pVT. The gene expression cassette for the cell-surface display of enhanced green fluorescent protein (eGFP) involved in the plasmid pleGFP-SSS (Inokuma et al. 2020) was amplified by PCR using the ScSED1p-F and ScSAG1t-R primers and then introduced into the *Sac*I site of pVT, resulting in the plasmid pVT-SceGFP.

The *IoSED1* coding region except for its secretion signal (Table S4) was amplified from *I. orientalis* NBRC1664 genomic DNA by PCR using the IoSED1a-F1 and IoSED1a-R1 primers. The amplified fragment was fused with the DNA fragment generated by PCR using Vector-F and Vector-R primers with plasmid plBG-SSS (Inokuma et al. 2016) as a template. The resulting plasmid was named plBG-SSIoS, in which the anchoring domain of plBG-SSS was replaced with the *IoSED1* anchoring domain. The plasmid for the cell-surface display of eGFP with the *IoSED1* promoter, secretion signal, and anchoring domain was constructed as follows: The DNA fragment containing the *IoSED1* promoter and secretion signal was amplified from *I. orientalis* NBRC10737 genomic DNA by PCR using the IoSED1ps-F1 and IoSED1ps-R1 primers. The DNA fragment of eGFP coding region involved in pleGFP-SSS was amplified by PCR using the eGFP-F and eGFP-R primers. The DNA fragment encoding the *IoSED1* anchoring domain was amplified from plBG-SSIoS by PCR using the IoSED1a-F2 and IoSED1a-R2 primers. These three fragments were introduced into the *Sac*I site of pVT to generate the plasmid pVT-IoeGFP.

The integrative plasmid enabling gene introduction into the previously identified intergenic sites of chromosome 2 (IS2) (Lee et al. 2022) of *I. orientalis* NBRC1664 was constructed as follows: Approximately 1 kbp of DNA fragments upstream and downstream of the IS2 were amplified from *I. orientalis* NBRC1664 genomic DNA using the IS2-UP-F and IS2-UP-R primers and IS2-DOWN-F and IS2-DOWN-R primers, respectively. In the overlap region of IS2-UP-R, a *Pac*I recognition site was inserted. These two DNA fragments were fused via overlap extension PCR to obtain the DNA fragment containing one *Pac*I site in the center. This fragment was introduced into the *Ppu*MI site of pVT, and the resulting plasmid was named pVT-IS. The gene expression cassette for displaying eGFP involved in pVT-IoeGFP was amplified by PCR using IoSED1p-F and ScSAG1t-R primers, and the resulting fragment was introduced into the *Sac*I site of pVT-IS. The resulting plasmid was named pVT-IS-IoeGFP.

The integrative plasmid for the cell-surface display of *A. aculeatus* BGL1 was constructed as follows: DNA fragments of the *IoSED1* promoter and secretion signal and the *IoSED1* anchoring domain were amplified from pVT-IoeGFP using the IoSED1ps-F1 and IoSED1ps-R2 primers and IoSED1a-F2 and IoSED1a-R2 primers, respectively. The DNA fragment encoding *A. aculeatus* BGL1 was amplified from plBG-SSS by PCR using the BGL-F and BGL-R primers. These three fragments were introduced into the *Sac*I site of pVT-IS. The resulting plasmid was named pVT-IS-BGL.

### Construction of cell-surface display strains

To construct the *I. orientalis* strain displaying eGFP, the episomal plasmids carrying the eGFP-display cassette, pVT-SceGFP or pVT-IoeGFP, were introduced into the NBRC1664/ura3Δ strain, resulting in the 16-ScGFP-p and 16-IoGFP-p strains, respectively. The empty vector pVT was similarly introduced into the NBRC1664/ura3Δ strain, resulting in the 16-pVT strain. For genomic integration of the eGFP-display cassette, the integrative plasmid pVT-IS-IoeGFP was linearized with *Pac*I and integrated into the IS2 of the NBRC1664/ura3Δ strain, yielding the 16-IoGFP-int strain. Similarly, for the construction of the *I. orientalis* strain displaying BGL, the integrative plasmid pVT-IS-IoBGL was linearized with *Pac*I and integrated into the IS2 of the NBRC1664/ura3Δ strain, yielding the 16-IoBG-int strain.

All *I. orientalis* strains were transformed using the lithium acetate method as described previously (Xiao et al. 2014) with minor modifications. Yeast cells were cultured in 3 mL of YPD medium at 30 °C and 200 rpm overnight. An aliquot was then inoculated into 3 mL of fresh YPD medium with an initial OD_600_ of 0.2 and grown under the same conditions until the OD_600_ reached 1.0. Cells were collected by centrifugation at 14,010 × *g* for 2 min and washed once with 0.1 M lithium acetate (LiAc). The cells were resuspended in 1 mL of 0.1 M LiAc and incubated at 30 °C for 15 min. After removing the supernatant, the pellet was mixed with 25 µL of yeast carrier DNA (TaKaRa), 36 µL of 1 M LiAc, 50 µL of intact or linearized plasmid DNA solution, and 240 µL of 50% (w/v) polyethylene glycol 4000. The mixture was incubated at 30 °C for 30 min, followed by the addition of 40 µL of dimethyl sulfoxide and heat shock treatment at 42 °C for 1 h. The cells were collected by centrifugation at 14,010 × *g* for 2 min, washed once with YPD medium, and resuspended with 1 mL of YPD medium. After recovery at 30 °C and 200 rpm for 2 h, the cells were spread on the appropriate agar plate for selection.

### Evaluation of eGFP expression

A single colony of 16-pVT, 16-ScGFP-p, 16-IoGFP-p, and 16-IoGFP-int was inoculated into 3 mL of SC-URA medium with 0.1 M 3-[N-morpholino] propanesulfonic acid (MOPS) buffer (pH 6.5) and cultivated at 30 °C and 200 rpm for 72 h. Then, the cells were transferred to 5 mL of the same fresh medium with an initial OD_600_ of 0.05 and cultivated at 30 °C and 200 rpm for 24 h. After cultivation, the cells were collected by centrifugation at 1000×*g* for 5 min and washed twice with Dulbecco’s phosphate-buffered saline (Nacalai Tesque, Kyoto, Japan). The washed cells were observed using a fluorescence microscope BZ-X810 (Keyence, Osaka, Japan) equipped with a Nikon Plan Apo λ 100×/1.45 oil-immersion objective lens (Nikon, Tokyo, Japan) and appropriate filters for eGFP. In addition, the washed cells were also subjected to flow cytometric analysis under 488-nm excitation using a SA3800 Cell Analyzer (Sony, Tokyo, Japan).

### Enzyme assays

For evaluation of BGL activity, p-nitrophenyl-β-D-glucopyranoside (pNPG) was used as a substrate, following the previously reported method (Inokuma et al. 2014). A single colony of NBRC1664/ura3Δ, 16-IoBG-int, and BY-BG-SSS, an *S. cerevisiae* strain that displays *A. aculeatus* BGL1 on its cell surface (Inokuma et al. 2016), was inoculated into 3 mL of YPD medium and cultivated at 30 °C and 200 rpm for 24 h. Then, the cells were transferred to 50 mL of YPD medium in a 200-mL flask with an initial OD_600_ of 0.05 and cultivated at 30 °C and 150 rpm for 72 h. The cells were separated from culture supernatants by centrifugation at 1000 × *g* for 5 min, and the supernatants were collected. The cell pellet was washed twice with Milli-Q water and then resuspended in Milli-Q water with the OD_600_ of 4. Then, 100 µL of the suspended cell solution or culture supernatant was added to 400 µL of 2.5 mM pNPG solution dissolved in 50 mM sodium citrate buffer (pH 5.0). After incubation at 30 °C or 45 °C and 500 rpm for 10 min in a shaker incubator M-BR-022UP (Taitec, Saitama, Japan), 500 µL of 3 M sodium carbonate solution was added to terminate the reaction. The absorbance of p-nitrophenol liberated by BGL activity was measured at 400 nm. The molar extinction coefficient of 18,720 M^−1^ cm^−1^ was used to calculate the concentration of p-nitrophenol in the assay mixture. One unit of enzyme activity was defined as the amount of enzymes required to liberate 1 µmol of p-nitrophenol per minute.

### Culture with cellobiose as the sole carbon source

A single colony of 16-IoBG-int and BY-BG-SSS was pre-cultured in 3 mL of YPD medium at 30 °C and 200 rpm for 48 h. The pre-culture was then inoculated into 5 mL of SC medium containing 1.9% cellobiose instead of glucose with 0.1 M MOPS (pH 6.5) in an L-shaped test tube with an initial OD_600_ of 0.05. Cultivation was performed using a TVS062CA shaker at 30 °C and 40 rpm for 72 h. For growth assays in the presence of lignocellulosic fermentation inhibitors, SC medium with 0.1 M MOPS containing acetic acid (final concentration, 100 mM; pH 4.0) or 5-HMF (final concentration, 25.4 mM; pH 6.5) was used.

The concentration of cellobiose in the medium was determined by high-performance liquid chromatography (HPLC). A Shimadzu prominence liquid chromatograph (Shimadzu, Kyoto, Japan) equipped with a CBM-20A system controller, a LC-20AB binary pump, a SIL-20AC autosampler, a RID-20A refractive index detector, a CTO-20AC oven, and a DGU-20A degasser was employed. An HPX-87H column (BioRad, CA, USA) was used, and the HPLC system was operated with 5 mM sulfuric acid as the mobile phase at a flow rate of 0.6 mL/min and a column temperature of 65 °C.

## Result

### Evaluation of the stress tolerance of *I. orientalis* NBRC1664

**Fig. 1:**
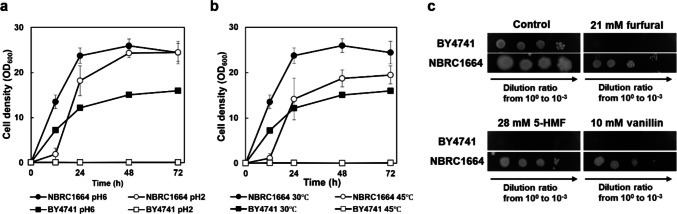
Characterization of the multi-stress tolerance of *I. orientalis* NBRC1664. Growth under (**a**) the acidic conditions and (**b**) the high-temperature conditions was evaluated in SC medium. *S. cerevisiae* BY4741 was used as a reference. All data represent the mean ± standard deviation of three independent experiments. **c** Growth in the presence of lignocellulosic fermentation inhibitors. Serial dilutions of cell suspensions (OD_600_ of 1, 0.1, 0.01, or 0.001) were spotted onto SD agar plates and incubated at 30 °C. A representative result from two independent experiments is shown

To compare the multi-stress tolerance of *I. orientalis* with that of the model yeast *S. cerevisiae*, we evaluated the stress tolerance of *I. orientalis* NBRC1664, the most tolerant among 11 *I. orientalis* strains obtained from NBRC (Fig. S1), and *S. cerevisiae* BY4741 under low pH, high temperature, and lignocellulosic fermentation inhibitors. As shown in Fig. 1a, both species grew well at pH 6, even though *I. orientalis* exhibited better growth than *S. cerevisiae*. *I. orientalis* showed robust growth at pH 2, with an OD_600_ after 72 h being nearly identical to that observed at pH 6, whereas *S. cerevisiae* failed to grow at pH 2. Similarly, *I. orientalis* successfully grew but *S. cerevisiae* did not at 45 °C; both strains grew well at the normal temperature of 30 °C (Fig. 1b). Furthermore, to evaluate growth in the presence of the fermentation inhibitors (21 mM furfural, 28 mM 5-HMF, and 10 mM vanillin), spot assays were performed (Fig. 1c). The concentrations of each inhibitor used in this study fall within the range observed in lignocellulosic hydrolysate (Mills et al. 2009). Both species showed distinct colony formation under non-stress conditions on SD medium supplemented with the appropriate nutrients. When exposed to the fermentation inhibitors, *I. orientalis* successfully formed clear colonies under all three inhibitor conditions, whereas *S. cerevisiae* failed to grow under any of the inhibitor conditions. These results are consistent with those reported by Akita and Matsushika (2024). Collectively, these results suggested that *I. orientalis* NBRC1664 possessed remarkable multi-stress tolerance.

### Evaluation of eGFP-displaying *I. orientalis* strains

**Fig. 2:**
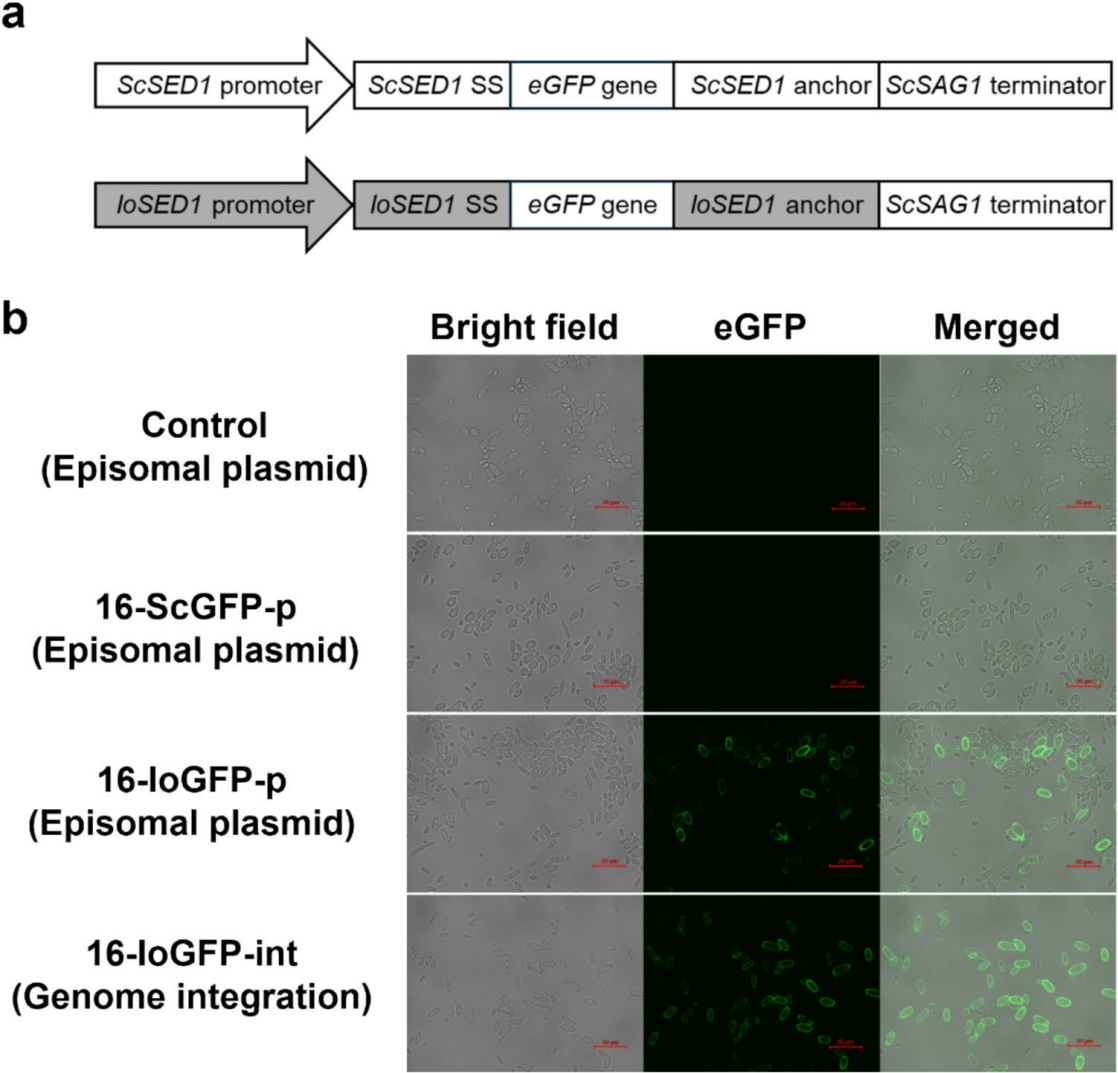
Fluorescence microscopy of strains displaying eGFP. **a** Schematic representation of the constructed cell-surface display cassettes for displaying eGFP. **b** Fluorescence images of eGFP-displaying strains. The cells were cultivated in SC-URA medium supplemented with 0.1 M MOPS buffer (pH 6.5) for 24 h. SS, secretion signal; anchor, anchoring domain; *Sc*, *Saccharomyces cerevisiae*; *Io*, *Issatchenkia orientalis*

To determine whether proteins fused with GPI-anchoring domains could be immobilized on the cell surface of *I. orientalis*, we constructed eGFP-display gene expression cassettes and examined the localization of the eGFP signal by fluorescence microscopy. First, we tried to express the gene expression cassette previously shown to display eGFP on the cell surface of *S. cerevisiae* (Inokuma et al. 2020), consisting of the *S. cerevisiae SED1* (*ScSED1*) promoter, secretion signal, and anchoring domain, together with the *S. cerevisiae SAG1* terminator (Fig. 2a). The episomal plasmid harboring this cassette (pVT-SceGFP) was introduced into *I. orientalis* NBRC1664/ura3Δ strain, a uracil-auxotrophic strain whose uracil auxotrophy was confirmed by growth analysis (Fig. S2), and fluorescence microscopy was conducted as described in the “Materials and methods” section. However, there was no detectable eGFP signal (Fig. 2b).

Next, we hypothesized that the lack of fluorescence resulted from poor function of the heterologous genetic elements within the cassette in *I. orientalis*. The promoter, secretion signal, and anchoring domain of the cell-surface display cassette critically influence display efficiency (Inokuma et al. 2014, 2016; Zhang et al. 2013). Therefore, we constructed a modified display cassette in which each of these elements was replaced with the corresponding element from the *IoSED1* gene (Fig. 2a), which is homologous to the *ScSED1* gene (Kitagawa et al. 2010) and is predicted to code a GPI-anchored protein based on prediction tools such as big-PI Fungal Predictor (Eisenhaber et al. 2004). Additionally, the signal sequence derived from the *IoSED1* gene has already been reported to function in mediating the extracellular secretion of target proteins (Kitagawa et al. 2010). This newly constructed display cassette was introduced into NBRC1664/ura3Δ, and fluorescence microscopy revealed distinct surface-localized fluorescence (Fig. 2b). The fluorescence pattern was comparable to that observed in the eGFP-displaying *S. cerevisiae* strain (Inokuma et al. 2020), suggesting successful display of eGFP on the cell surface of *I. orientalis*.

Although a surface-localized eGFP signal was detected, fluorescence was observed in only a limited number of cells. To increase the proportion of cells displaying eGFP, we integrated the display cassette into the genome. An integrative plasmid designed to insert the cassette into the IS2 (Lee et al. 2022) of *I. orientalis* NBRC1664 was constructed as described in the “Materials and methods” section, and the eGFP-display cassette was integrated into the NBRC1664/ura3Δ strain. Compared with the plasmid-harboring strain (16-IoGFP-p), fluorescence microscopy of the genome-integrated strain (16-IoGFP-int) revealed a much larger proportion of cells showing a clear surface-localized signal (Fig. 2b), which is consistent with the flow cytometry results (Fig. S3). Taken together, these results demonstrated the successful construction of a gene expression cassette that enabled the display of target proteins on the cell surface of *I. orientalis*.

### Evaluation of *I. orientalis* strains displaying β-glucosidase

**Fig. 3:**
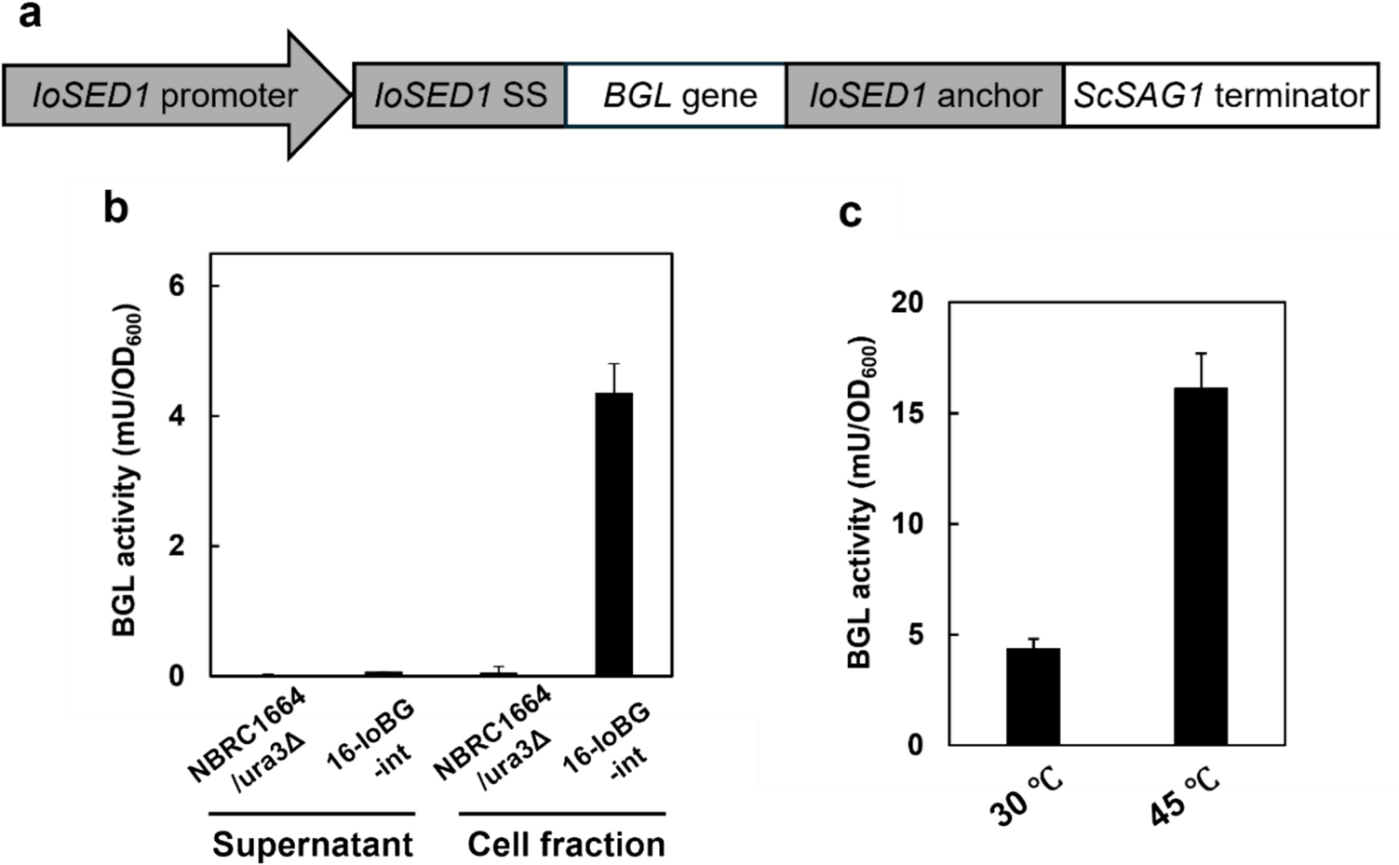
Enzyme activity assay of BGL-displaying strains. **a** Schematic representation of the constructed cell-surface display cassette for displaying BGL. **b** Evaluation of BGL activity of the BGL-displaying *I. orientalis* strain (16-IoBG-int). Its parental strain NBRC1664/ura3Δ was used as a control. The cells were cultivated in YPD medium for 72 h, and the culture supernatant and cell fractions were individually subjected to the BGL activity assay. **c** Evaluation of BGL activity of 16-IoBG-int under the high-temperature conditions. All data represent the mean ± standard deviation of three independent experiments

Next, using the constructed gene expression cassette for protein cell-surface display, we constructed an *I. orientalis* strain displaying *A. aculeatus* BGL1, which hydrolyzes cellobiose into glucose (Murao et al. 1988), yielding the 16-IoBG-int strain (Fig. 3a). The strain was cultured in YPD medium at 30 °C for 72 h, after which BGL activity in the cell fraction and culture supernatant was evaluated at 30 °C. No detectable BGL activity was observed in the culture supernatant of either 16-IoBG-int or its parental strain. In contrast, the cell fraction of 16-IoBG-int exhibited significantly higher BGL activity (4.35 ± 0.5 mU/OD_600_), whereas that of the parental strain exhibited almost no detectable activity (Fig. 3b). This indicated the successful cell-surface display of BGL in *I. orientalis*.

Additionally, to evaluate the activity of the displayed *A. aculeatus* BGL1 under the high-temperature conditions expected during LCB bioconversion (Yanase et al. 2010), the cells cultured at 30 °C for 72 h were subjected to the same BGL activity assay at 45 °C. When performing at 45 °C, BGL activity markedly increased to more than threefold higher (16.1 ± 1.6 mU/OD_600_) compared to that measured at 30 °C (Fig. 3c). This result is consistent with the fact that the optimal temperature of *A. aculeatus* BGL1 lies in the high-temperature range above 50 °C (Murao et al. 1988).

### Fermentation with cellobiose as the sole carbon source under stress conditions

**Fig. 4:**
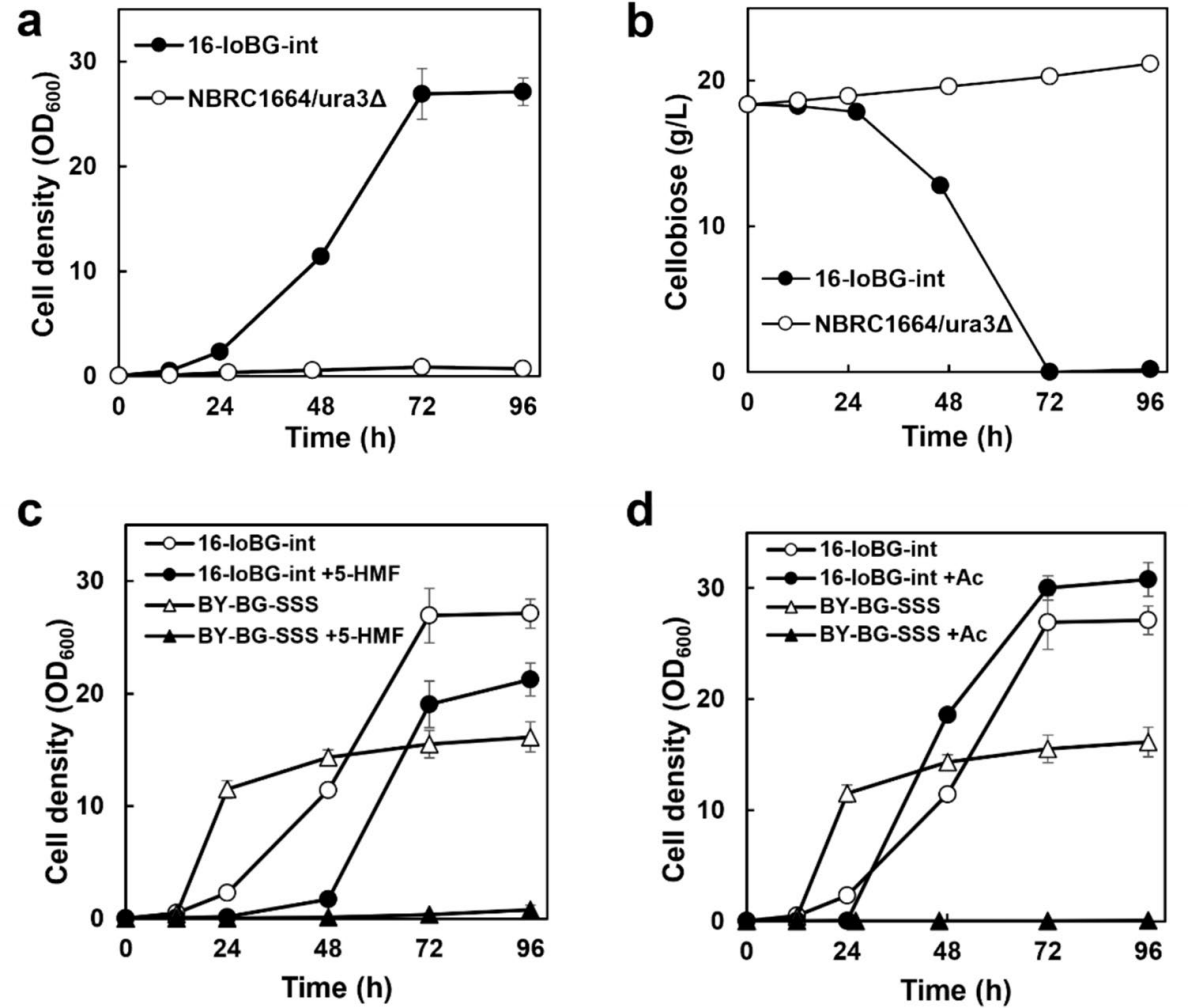
Growth of the BGL-displaying strain on cellobiose as the sole carbon source. **a** Cell growth and (**b**) residual cellobiose concentration during cultivation in SC medium containing 1.9% cellobiose and 0.1 M MOPS (pH 6.5). Growth of the strains in the same medium supplemented with (**c**) 25.4 mM 5-HMF or (**d**) 100 mM acetic acid. All data are presented as the mean ± standard deviation of three independent experiments. Ac, acetic acid

To evaluate whether the constructed BGL-displaying strain 16-IoBG-int could hydrolyze the disaccharide cellobiose and utilize the released glucose, the strain was cultivated in SC medium containing 1.9% cellobiose as the sole carbon source. The strain consumed cellobiose efficiently and grew robustly, completely depleting the substrate after 72 h (Fig. 4 a and b). In contrast, the parental strain failed to grow under the same conditions. These results demonstrated that 16-IoBG-int degraded cellobiose via cell surface–displayed BGL and utilized the released glucose for its growth.

Additionally, we investigated whether 16-IoBG-int could assimilate cellobiose in the presence of lignocellulosic fermentation inhibitors. The strain was cultivated in the same SC medium containing 1.9% cellobiose as the sole carbon source, supplemented with either acetic acid or 5-HMF. As a reference, we used BY-BG-SSS, an *S. cerevisiae* strain displaying the same *A. aculeatus* BGL1 on its cell surface, which exhibits high BGL activity (Inokuma et al. 2016). As shown in Fig. 4 c and d, both strains exhibited robust growth in the absence of inhibitors, with *S. cerevisiae* showing higher OD_600_ than *I. orientalis* during the first 48 h of cultivation. In the presence of 25.4 mM 5-HMF, *S. cerevisiae* failed to grow, whereas *I. orientalis* exhibited clear growth, although its OD_600_ was lower than that under inhibitor-free conditions. Similarly, in the presence of 100 mM acetic acid, no growth of *S. cerevisiae* was observed, whereas *I. orientalis* showed even higher growth than in the absence of inhibitors. Additionally, we evaluated the assimilation of cellobiose at 45 °C. However, in contrast to the robust growth observed for the wild-type at the same temperature (Fig. 1b), 16-IoBG-int did not grow (Fig. S4). Collectively, these results demonstrated that the *I. orientalis* strain displaying BGL assimilated cellobiose even under certain stress conditions.

## Discussion

In this study, we developed a cell-surface display cassette applicable to the multi-stress-tolerant yeast *I. orientalis*. Using this cassette, we successfully constructed a strain displaying *A. aculeatus* BGL1 on its cell surface that exhibited robust growth on cellobiose as the sole carbon source. Furthermore, this strain was able to assimilate cellobiose under several stress conditions associated with LCB bioconversion, suggesting that *I. orientalis* strains with cellulolytic enzymes are well suited for efficient LCB bioconversion.

We constructed a new gene expression cassette by employing the promoter, secretion signal, and anchoring domain derived from the *IoSED1* gene and showed the successful display of eGFP (Fig. 2b). We found that genome integration of the cassette markedly increased the proportion of cells exhibiting eGFP fluorescence on the cell surface compared with the episomal plasmid-based expression system, suggesting that plasmid-based expression was intrinsically unstable in *I. orientalis* NBRC1664. This interpretation is supported by a previous report showing that episomal plasmids carrying the same *S. cerevisiae*–derived centromere and autonomously replicating sequence exhibit instability in another *I. orientalis* strain (Tran et al. 2019; Cao et al. 2020). On the other hand, even in the genome-integrated strain, a subset of non-fluorescent cells was still observed (Figs. 2b and S3). Notably, as shown in Fig. 2b, cells with smaller sizes exhibited little or no fluorescence, suggesting that the introduced cassette did not function in these cells. According to Shimoi et al. (1998), the *Sc*Sed1 protein is present at relatively low abundance in the cell wall during the exponential growth phase but becomes the most abundant cell wall protein during the stationary phase in *S. cerevisiae*. Given that *IoSED1* shares homology with *ScSED1*, its expression might also be suppressed during the exponential phase, leading to reduced *IoSED1* promoter activity in the smaller and actively growing cells of 16-IoGFP-int. Although the *IoSED1* promoter is among the strongest in *I. orientalis* (Cao et al. 2020), its temporal expression dynamics have not been characterized, warranting further investigation.

Using the newly constructed gene expression cassette, we successfully obtained an *I. orientalis* strain that displayed *A. aculeatus* BGL1 on its cell surface (16-IoBG-int). However, when the *S. cerevisiae* BY-BG-SSS displaying the same *A. aculeatus* BGL1 (Inokuma et al. 2016) was subjected to the same BGL activity assay in this study, it exhibited BGL activity of 72 ± 1.4 mU/OD_600_ (Fig. S5), approximately 20-fold higher than that of 16-IoBG-int, indicating that further improvement of the display system in *I. orientalis* is required. The activity of the surface-displayed BGL is affected by the promoter, secretion signal, and anchoring domain incorporated in the cassette, and optimization of these individual elements has been reported in other yeast species (Zhang et al. 2013; Inokuma et al. 2016; Phienluphon et al. 2019). In particular, the anchoring domain determines the localization of the fused functional protein within the cell wall, thereby affecting its accessibility to extracellular substrates and, consequently, its activity (Inokuma et al. 2020, 2023). Given that *Io*Sed1 is currently the only anchoring domain available for *I. orientalis*, future work should include a comprehensive screening of anchoring domains in this yeast, following the systematic analyses previously performed for other yeast species. Another possible explanation for the low BGL activity is the difference in post-translational modification. Crystallographic analysis by Suzuki et al. (2013) revealed that *A. aculeatus* BGL1 is highly glycosylated with 88 monosaccharides in the dimer. Because glycosylation plays critical roles in protein secretion and stability (Hou et al. 2012; Yang et al. 2015), and species-specific differences in glycosylation among yeasts have been documented (Karbalaei et al. 2020), it is conceivable that glycosylation of *A. aculeatus* BGL1 in *I. orientalis* impaired its secretion or stability, thereby lowering its enzymatic activity. Taken together, further analyses are required to identify key factors for improving BGL activity, such as quantifying the amount of surface-displayed BGL (e.g., via antibody staining) and comparing glycosylation patterns between host species. Based on these findings, future efforts will focus on optimizing each component of the display cassette or exploring alternative BGLs derived from other microorganisms.

The BGL-displaying strain was able to assimilate cellobiose even in the presence of acetic acid and 5-HMF, which are major lignocellulosic fermentation inhibitors (Jönsson and Martín, 2016) (Fig. 4 c and d). In particular, in the presence of 100 mM acetic acid, the strain showed robust growth, and notably, its OD_600_ after 96 h was even higher than that under acetic acid–free conditions (Fig. 4d). This was likely due to acetic acid assimilation, as previously reported by Jeong et al. (2025). Such acetic acid consumption is highly desirable, as lignocellulosic hydrolysates typically contain substantial amounts (10–15 g/L) of acetic acid (Du et al. 2024). In contrast, this strain failed to assimilate cellobiose at 45 °C. Considering that the wild-type strain could grow at 45 °C (Fig. 1b) and that the surface-displayed *A. aculeatus* BGL1 showed high hydrolytic activity at the same temperature (Fig. 3c), it is likely that the fusion protein comprising *A. aculeatus* BGL1 and the *Io*Sed1 anchoring domain might not be properly immobilized on the cell surface under the high-temperature conditions. High-temperature conditions induce misfolding of heterologous proteins in the endoplasmic reticulum, reducing secretion in the methylotrophic yeast *Komagataella phaffii* (Dragosits et al. 2009; Zhong et al. 2014). In addition, misfolded proteins suppress cell growth (Geiler-Samerotte et al. 2011). Thus, elevated temperatures may have reduced the folding efficiency of heterologous proteins in *I. orientalis*, leading to the accumulation of misfolded fusion proteins and growth inhibition. Another possible explanation is insufficient hydrolytic activity of the surface-displayed BGL to overcome high-temperature stress. Under heat stress, yeast cells exhibit dynamic transcriptional responses, including upregulation of genes encoding heat shock proteins that prevent protein aggregation and misfolding and desaturases that promote the synthesis of unsaturated fatty acids (Lu et al. 2022; Miller et al. 1979; Postmus et al. 2008). These responses collectively increase cellular energy demand. Given that an increased glycolytic flux under heat stress also occurs in *I. orientalis* (Frousnoon et al. 2025), it is conceivable that enhanced ATP generation is required for thermal adaptation in this yeast as well. In the present study, with cellobiose as the sole carbon source, ATP production depended on glucose supplied through cellobiose hydrolysis by the surface-displayed BGL. Thus, even though the hydrolytic activity of the displayed BGL increases (Fig. 3c) and glucose supply from cellobiose is expected to be improved under the high-temperature conditions, the hydrolytic activity may still be insufficient to meet the elevated ATP demand at 45 °C, leading to impaired thermal adaptation and growth suppression. Taken together, future studies should verify proper cell-surface immobilization of BGL during cultivation at 45 °C and investigate whether increasing the hydrolytic activity of the displayed cellulase confers growth capability at the elevated temperature.

In conclusion, we successfully developed a cell-surface display system that enabled the display of target proteins on the surface of the multi-stress-tolerant yeast *I. orientalis*. To the best of our knowledge, this is the first report demonstrating the immobilization of functional proteins on the cell surface of this organism. This achievement provides a solid foundation for establishing *I. orientalis* as a robust microbial platform for cost-effective LCB bioconversion.

## Supplementary Information

Below is the link to the electronic supplementary material.

ESM 1 (PDF 732 KB)

## Data Availability

All data generated or analyzed during this study are included in this published article or its supplementary information file.
