# Supplementary material for: Engineering a robust cell-surface display platform in the multi-stress-tolerant yeast Issatchenkia orientalis
Source: Appl Microbiol Biotechnol. 2026 May 19;110:210. doi: 10.1007/s00253-026-13867-1 (PMC13357452; doi:10.1007/s00253-026-13867-1)
Supplement: Supplementary file 1 [file 253_2026_13867_MOESM1_ESM.pdf]

Applied Microbiology and Biotechnology – Supplementary material

Title:

Engineering a robust cell-surface display platform in the multi-stress-tolerant yeast *Issatchenkia orientalis*

Authors:

Yoshiaki Kawahara[1], Ryo Nasuno[2], Yong-Su Jin[3,4], Tomohisa Hasunuma[1,2,*]

Author Affiliations:

[1] Graduate School of Science, Technology and Innovation, Kobe University, 1-1 Rokkodai, Nada, Kobe 657-8501, Japan

[2] Engineering Biology Research Center, Kobe University, 1-1 Rokkodai, Nada, Kobe, 657-8501, Japan

[3] Department of Food Science and Human Nutrition, University of Illinois at Urbana−Champaign, Urbana, IL, 61801, USA

[4] Carl R. Woese Institute for Genomic Biology, University of Illinois at Urbana-Champaign, Urbana, IL, 61801, USA

* Corresponding author

Corresponding author:

Tomohisa Hasunuma

E-mail: hasunuma@port.kobe-u.ac.jp

Data Availability Statement

For plasmids and all other reagent requests, please contact the corresponding author.

Supplementary Figures

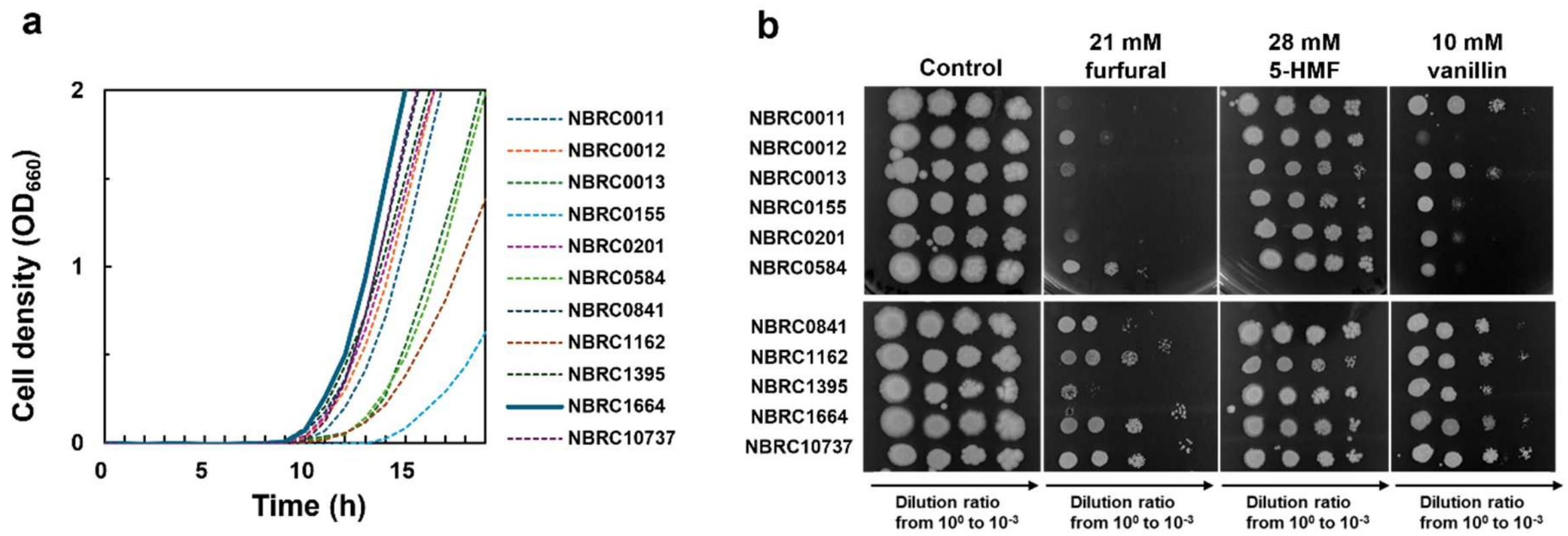

Fig. S1 Characterization of the multi-stress tolerance of 11 *I. orientalis* strains obtained from NITE Biological Resource Center (NBRC). (a) Growth under the acidic conditions (pH2) was evaluated in synthetic complete (SC) medium. Data represents the mean of three biological replicates. (b) Growth in the presence of lignocellulosic fermentation inhibitors. Serial dilutions of cell suspensions (OD_{600} of 1, 0.1, 0.01, or 0.001) were spotted onto synthetic dextrose (SD) agar plates and incubated at 30 °C. A representative result from two independent experiments is shown.

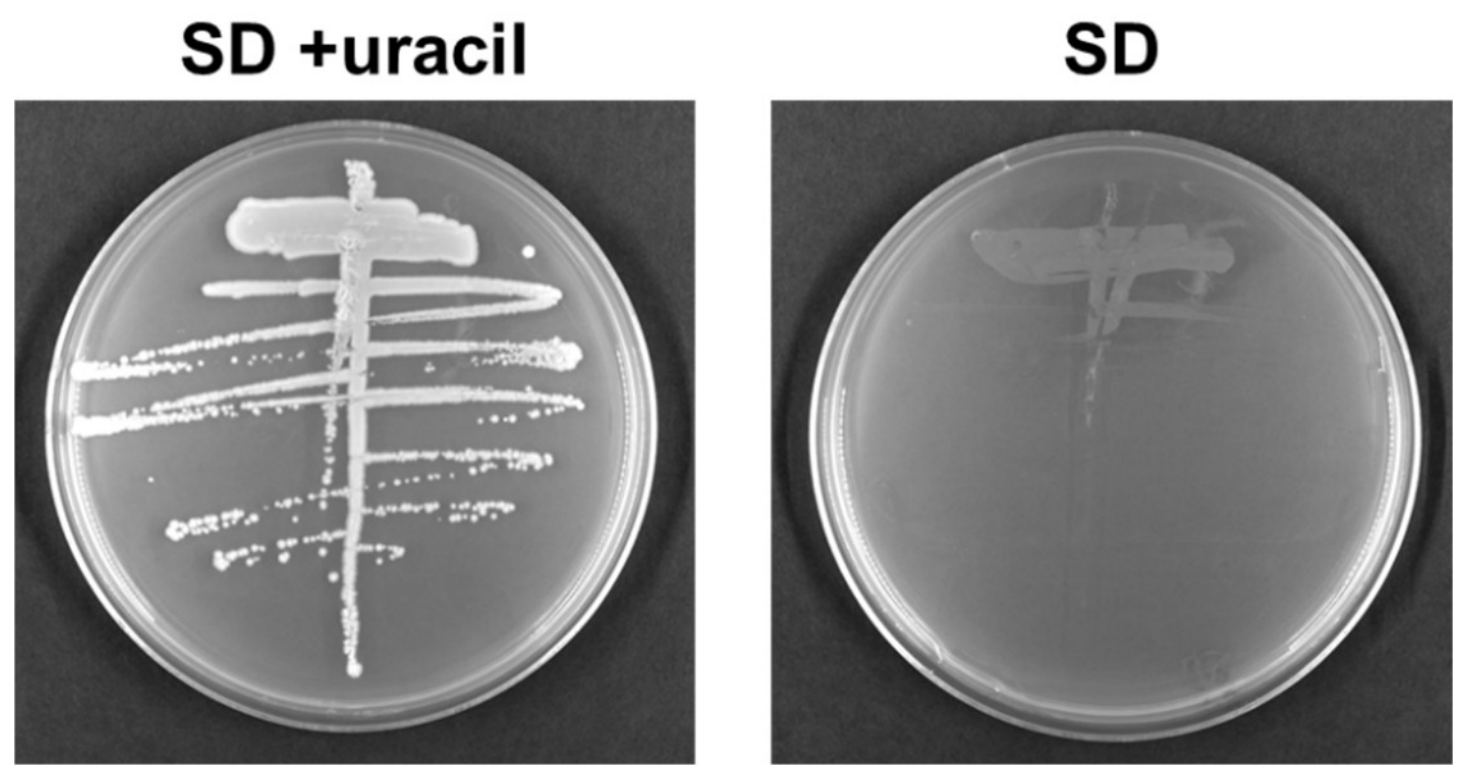

Fig. S2 Uracil auxotrophy of *I. orientalis* NBRC1664/ura3Δ. The uracil auxotrophic strain NBRC1664/ura3Δ was streaked onto SD agar medium with (left) or without (right) uracil supplementation and incubated at 30 °C.

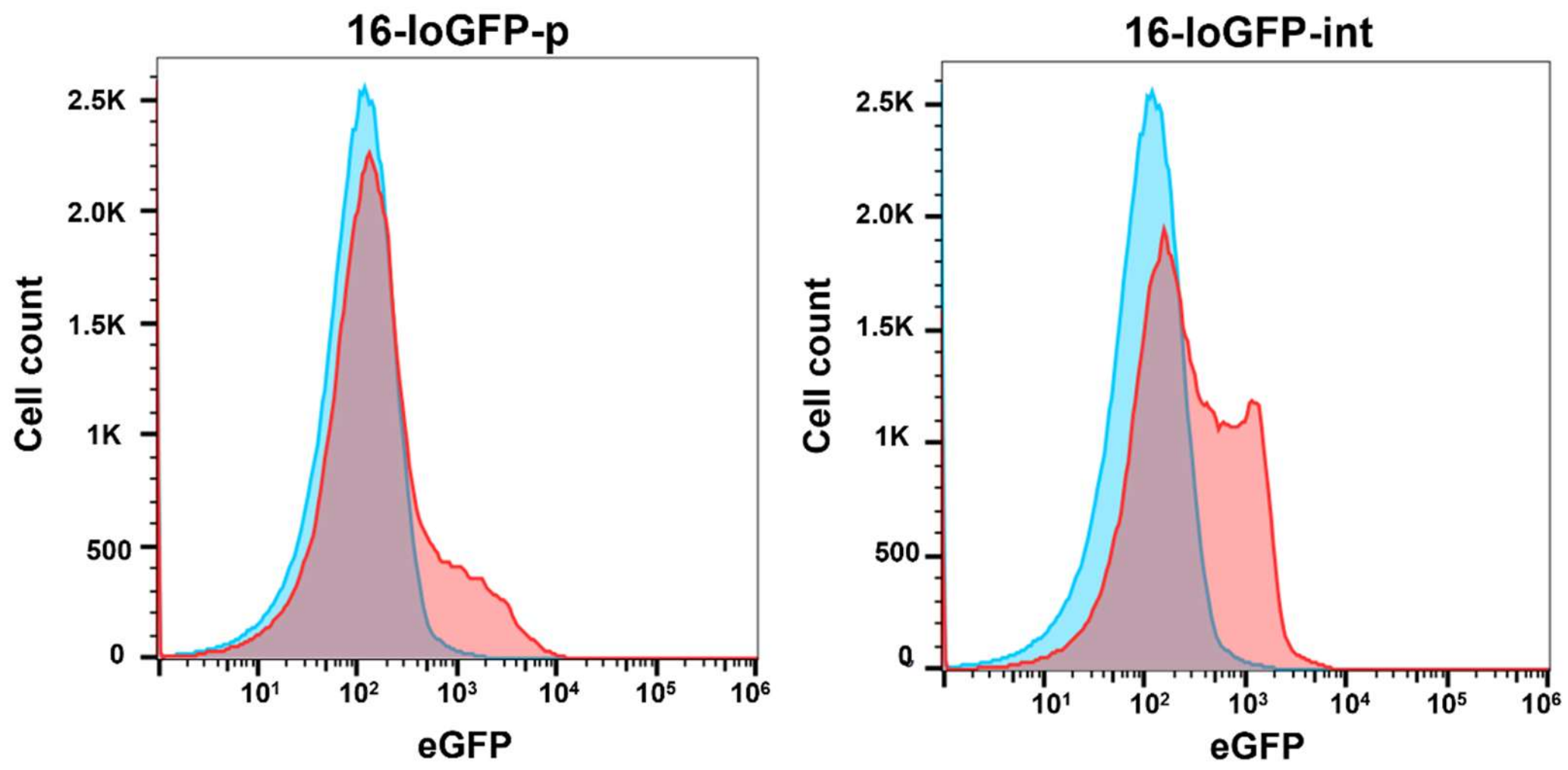

Fig. S3 Flow cytometric analysis of enhanced green fluorescent protein (eGFP)-displaying strains. eGFP expression profiles of the plasmid-harboring strain (16-IoGFP-p, left) and the genome-integrated strain (16-IoGFP-int, right) are shown. A representative result from three independent experiments is shown. The area filled in blue indicates the control strain (16-pVT) harboring the empty vector, and the area filled in red indicates the strain carrying the eGFP-display cassette.

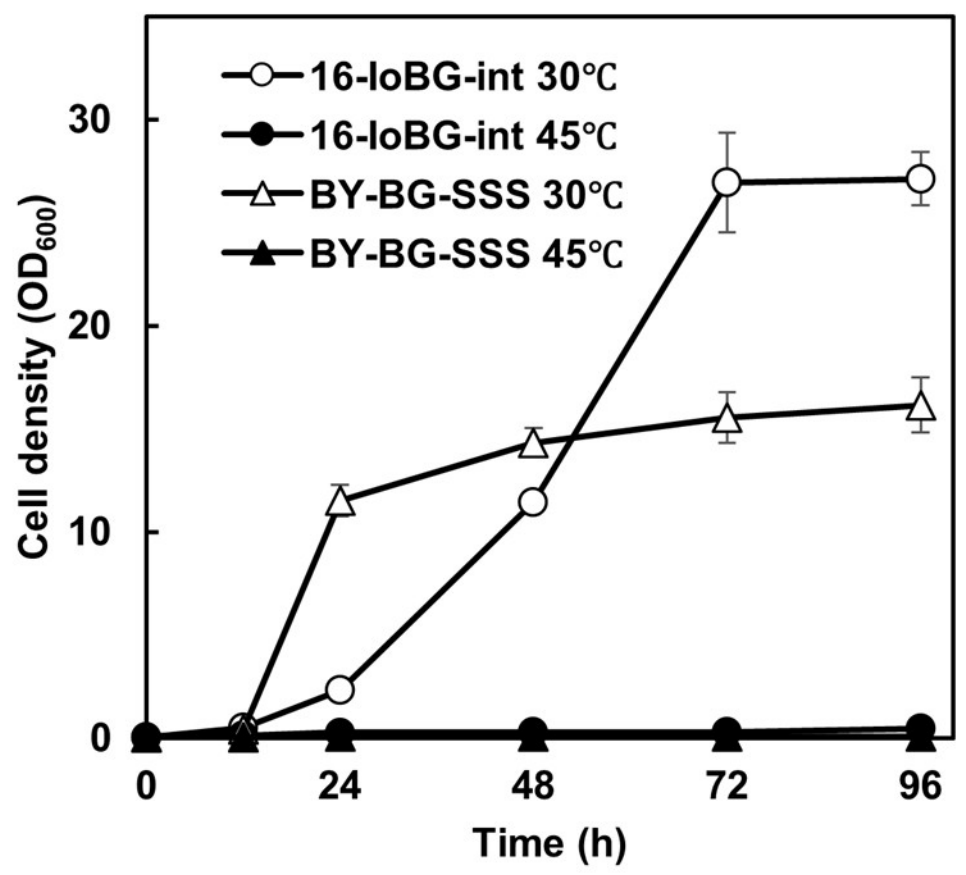

Fig. S4 Cultivation with cellobiose as the sole carbon source under the high temperature conditions. *I. orientalis* 16-IoBG-int and *S. cerevisiae* BY-BG-SSS were cultured at 45 °C in SC medium containing 1.9 % cellobiose and 0.1 M 3-[N-morpholino] propanesulfonic acid (pH 6.5). All data represent the mean ± standard deviation of three independent experiments.

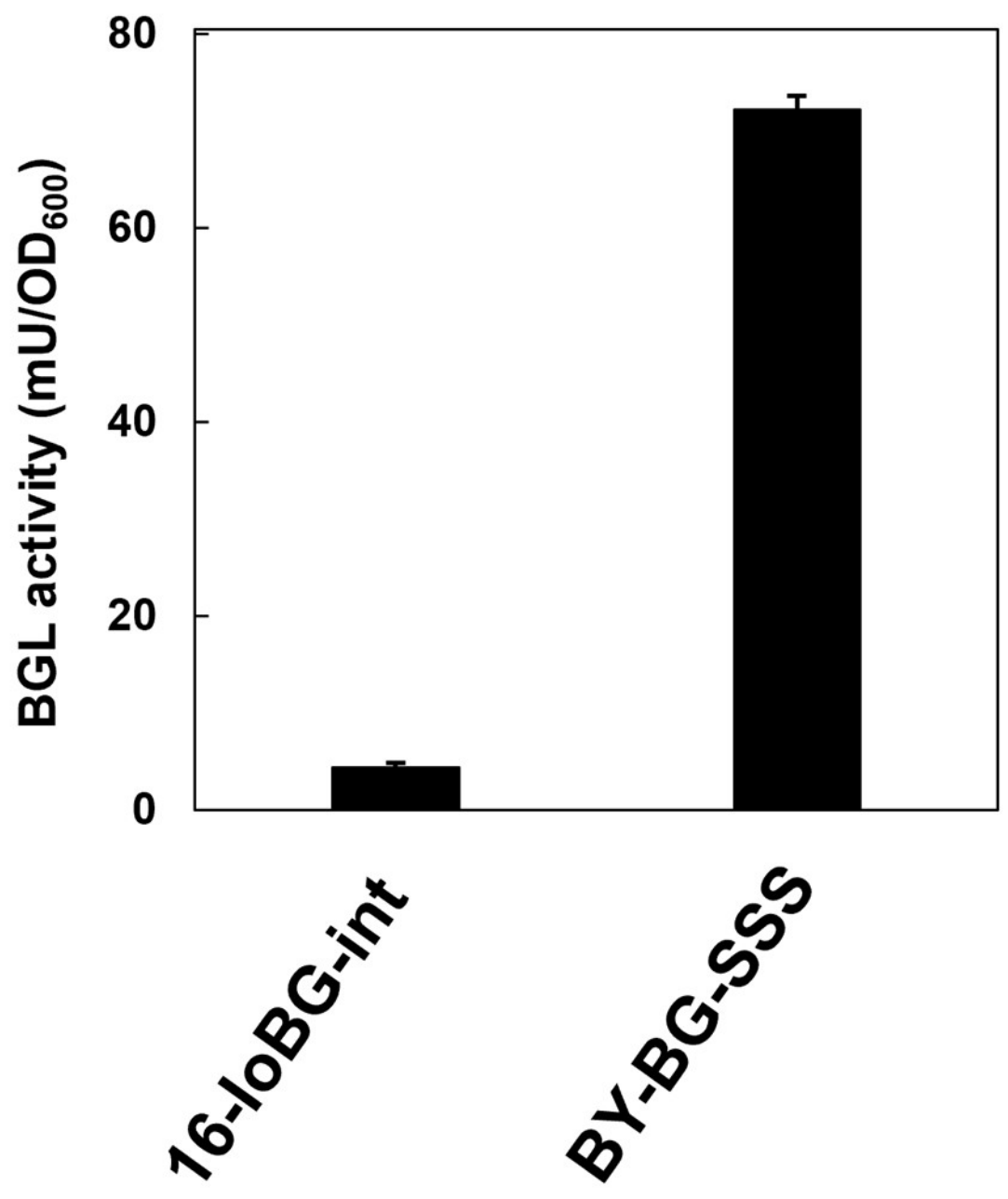

Fig. S5 β-Glucosidase (BGL) activity of *I. orientalis* 16-IoBG-int and *S. cerevisiae* BY-BG-SSS. All data represent the mean ± standard deviation of three independent experiments.

Supplementary Tables

Table S1 Strains used in this study.

Strain	Description	Source
S. cerevisiae		
BY4741	*MATa his3Δ1 leu2Δ0 met15Δ0 ura3Δ0*	Life Technologies
BY-BG-SSS	BY4741 strain carrying plBG-SSS	Inokuma et al. (2016)
I. orientalis		
NBRC0011	Wild type	NBRC
NBRC0012	Wild type	NBRC
NBRC0013	Wild type	NBRC
NBRC0155	Wild type	NBRC
NBRC0201	Wild type	NBRC
NBRC0584	Wild type	NBRC
NBRC0841	Wild type	NBRC
NBRC1162	Wild type	NBRC
NBRC1395	Wild type	NBRC
NBRC1664	Wild type	NBRC
NBRC10737	Wild type	NBRC
NBRC1664/ura3Δ	NBRC1664 strain lacking *ura3*	This study
16-pVT	NBRC1664/ura3Δ strain carrying pVT	This study
16-ScGFP-p	NBRC1664/ura3Δ strain carrying pVT-SceGFP	This study
16-IoGFP-p	NBRC1664/ura3Δ strain carrying pVT-IoeGFP	This study
16-IoGFP-int	NBRC1664/ura3Δ strain carrying pVT-IS-IoeGFP	This study
16-IoBG-int	NBRC1664/ura3Δ strain carrying pVT-IS-IoBGL	This study

Table S2 Plasmids used in this study. P, promoter; SS, secretion signal; A, anchoring domain; T, terminator.

Strain	Description	Source
pIBG-SSS	*ScHIS3, ScSED1*$_{P}$–*ScSED1*$_{SS}$–*A. aculeatus BGL1*–*ScSED1*$_{A}$–*ScSAG1*$_{T}$	Inokuma et al. (2016)
pIeGFP-SSS	*ScHIS3, ScSED1*$_{P}$–*ScSED1*$_{SS}$–*eGFP*–*ScSED1*$_{A}$–*ScSAG1*$_{T}$	Inokuma et al. (2020)
pIBG-SSIoS	*ScHIS3, ScSED1*$_{P}$–*ScSED1*$_{SS}$–*eGFP*–*IoSED1*$_{A}$–*ScSAG1*$_{T}$	This study
pRS415	*ScLEU2*	Stratagene
pVT	*ScLEU2, IoURA3*, pRS415 with *IoURA3*	This study
pVT-SceGFP	*ScLEU2, IoURA3*, pVT with *ScSED1*$_{P}$–*ScSED1*$_{SS}$–*eGFP*–*ScSED1*$_{A}$–*ScSAG1*$_{T}$	This study
pVT-IoeGFP	*ScLEU2, IoURA3*, pVT with *IoSED1*$_{P}$–*IoSED1*$_{SS}$–*eGFP*–*IoSED1*$_{A}$–*ScSAG1*$_{T}$	This study
pVT-IS	*IoURA3*, pVT with the sequence homologous to the regions flanking the IS2 site	This study
pVT-IS-IoeGFP	*IoURA3*, pVT-IS with *IoSED1*$_{P}$–*IoSED1*$_{SS}$–*eGFP*–*IoSED1*$_{A}$–*ScSAG1*$_{T}$	This study
pVT-IS-IoBGL	*IoURA3*, pVT-IS with *IoSED1*$_{P}$–*IoSED1*$_{SS}$–*A. aculeatus BGL1*–*IoSED1*$_{A}$–*ScSAG1*$_{T}$	This study

Table S3 Primers used in this study.

Name	Sequence
URA3-UP-F	AAACAGGGAAGGTTGACATT
URA3-UP-R	ATTTAAACTAGTGTACATGTATCAAATCTTTGTGTAAGAACCTTGACAAACAAACTACTT
URA3-DOWN-F	AGATCTTCAACGCTTTAATAAAGTAGTTTGTTTGTCAAGGTTCTTACACAAAGATTTGAT
URA3-DOWN-R	AACACTTAGAATACGCGGAA
Colony-P-F	CGGTATCGATAAGCTATTTGGTTCATTAACTTTTGGTTC
Colony-P-R	ATTCGATATCAAGCTTACCAAGAAGACGTTCATGTATG
URA3-F	CGGTATCGATAAGCTTAAACAGGGAAGGTTGACATTG
ScSED1p-F	CCGCCACCGCGGTGGATTGGATATAGAAAATTAACGT
ScSAG1t-R	GGGAACAAAAGCTGGTTTGATTATGTTCTTTCTATTTGA
IoSED1a-F1	AACCCGGGCCCGGGCCCTTCTCTAACGACACTGTTGTC
IoSED1a-R1	TGTACTAACTGTACATTATAATAAGTAGACAGCAGCACC
Vector-F	GGGCCCGGGCCCGGGTTGCACCTTCGGGAGCGCC
Vector-R	TGTACAGTTAGTACATTGAGTC
IoSED1ps-F1	AGCGGCCGCCACCGCGGTGGAATACACGAGAGATTATAGC
IoSED1ps-R1	CCTTGCTCACGAAAGCTGCGACAGCAGAACC
eGFP-F	CGCAGCTTTCGTGAGCAAGGGCGAGGAGC
eGFP-R	TGTCGTTAGAGGGCCCGGGCCCGGGCTTGTA
IoSED1a-F2	GCCCGGGCCCTCTAACGACACTGTTGTCACTG
IoSED1a-R2	CTAAAGGGAACAAAAGCTGGTTTGATTATGTTCTTTCTATTTG
IS2-UP-F	AGTGCCACCTGGGTCTTAAAGCATCAGAAATAAAAAG
IS2-UP-R	GGTAATCATGATACACGTATTTTAATTAAAGTATGTACGCAATAGGTG

IS2-DOWN-F	ATACGTGTATCATGATTACC
IS2-DOWN-R	ATATTATTTAAGGACTACAGACATAATACCTAC
IoSED1p-F	CCGCCACCGCGGTGGAATACACGAGAGATTATAGCAATAC
IoSED1ps-R2	CCAGTTCATCGAAAGCTGCGACAGCAGAACC
BGL-F	CGCAGCTTTCGATGAACTGGCGTTCTCTCCTCC
BGL-R	TGTCGTTAGAGGGCCCGGGCCCGGGTTGCACCTTC

Table S4 DNA sequences of each genetic element in the newly constructed display cassettes for *I. orientalis*.

Genetic element	Sequence
Promoter	aatacacgagagattatagcaatacacgagaaaccataagatactaaggtaaatcatggcaaatcatattaactattgacctctgacaaacagttatggccctttaaagaaggtaaaacgtgggaa gccttgggacaggaaaaaaaaaaaaaaccttctctctcaatgagccaacttttcattacatcatcatcgtccacgatttaattggacaataggaaaatgcaaaacaaataaagctgagtaaagag cggcaaaaatatgcaaaagagacaaagatttgccaaagaggcaaagatctgcagaaatgggaaaaaaaacttcataaattgcaaaacgcgcttctatttttagtacattcgccagcggccgtgctgt ttatcttttgccgcttacggaaggcgcgcgccgccggtggctgttttctggtaaagtgactcttccacggggggaagctataaaaagcgtgaaatccctcccacattttctaatcccagtggtaaccca cacttcttttctatagttttttagttttattctttttcacttatcaacttttatcgttcatagtctctcgcttacaaactaacacaataaaa
Secretion signal	atgcaattcaagtacttagcaccattagctttagcaggttctgctgtcgcagctttc
Anchoring domain	tctaacgacactgttgtcactgaagttgtcactgcttacaccacttactgcccagaaccaactgaaattacccaaaacggtaagacttacactgtcactgaagcaaccaccttgactatcactgactgtcc atgtaccatcacccacacctcctctgctgctccaacccacaacccagctccaaccactgcttctgaagaattctctgcaggtgcaggtaaggttggtgttgcaggtttagctgctgctgcaggtgctgc tgtctacttattataa
Terminator	tgtacagttagtacattgagtcgaaatatacgaaattattgttcataattttcatcctggctcttttttcttcaaccatagttaaatggacagttcatatcttaactctaataatacttttctagttcttatccttttcc gtctcaccgcagattttatcatagtattaaatttatattttgttcgtaaaaagaaaaatttgtgagcgttaccgctcgtttcattacccgaaggctgtttcagtagaccactgattaagtaagtagatgaaaaa atttcatcaccatgaaagagttcgatgagagctactttttcaaatgcttaacagctaaccgccattcaataatgttacgctctcttcattctgcggctacgttatctaacaaaaggttttactctctcatatc tcattcaaatagaaagaacataatcaaa
